# The Gut Microbial Composition Is Species-Specific and Individual-Specific in Two Species of Estrildid Finches, the Bengalese Finch and the Zebra Finch

**DOI:** 10.3389/fmicb.2021.619141

**Published:** 2021-02-19

**Authors:** Öncü Maraci, Anna Antonatou-Papaioannou, Sebastian Jünemann, Omar Castillo-Gutiérrez, Tobias Busche, Jörn Kalinowski, Barbara A. Caspers

**Affiliations:** ^1^Department of Behavioural Ecology, Bielefeld University, Bielefeld, Germany; ^2^Evolutionary Biology, Bielefeld University, Bielefeld, Germany; ^3^Institute of Biology-Zoology, Freie Universität Berlin, Berlin, Germany; ^4^Faculty of Technology, Bielefeld University, Bielefeld, Germany; ^5^Center for Biotechnology (CeBiTec), Bielefeld University, Bielefeld, Germany

**Keywords:** gut microbiota, symbionts, birds, zebra finch, Bengalese finch, host-specific factors, inter-individual differences, temporal stability

## Abstract

Microbial communities residing in the gastrointestinal tracts of animals have profound impacts on the physiological processes of their hosts. In humans, host-specific and environmental factors likely interact together to shape gut microbial communities, resulting in remarkable inter-individual differences. However, we still lack a full understanding of to what extent microbes are individual-specific and controlled by host-specific factors across different animal taxa. Here, we document the gut microbial characteristics in two estrildid finch species, the Bengalese finch (*Lonchura striata domestica*) and the zebra finch (*Taeniopygia guttata*) to investigate between-species and within-species differences. We collected fecal samples from breeding pairs that were housed under strictly controlled environmental and dietary conditions. All individuals were sampled at five different time points over a range of 120 days covering different stages of the reproductive cycle. We found significant species-specific differences in gut microbial assemblages. Over a period of 3 months, individuals exhibited unique, individual-specific microbial profiles. Although we found a strong individual signature in both sexes, within-individual variation in microbial communities was larger in males of both species. Furthermore, breeding pairs had more similar microbial profiles, compared to randomly chosen males and females. Our study conclusively shows that host-specific factors contribute structuring of gut microbiota.

## Introduction

Animal bodies are inhabited by billions of microorganisms, which are collectively termed microbiota. The majority of these microorganisms reside in the gastrointestinal tract (gut) of the animals (Qin et al., 2010). A strong body of evidence has left no doubt that several physiological processes of the animal hosts, which had been previously attributed to the host itself, are functionally influenced by microbial activities (Tremaroli and Bäckhed, 2012; McFall-Ngai et al., 2013; Kohl and Carey, 2016). Consequently, the microbial symbionts influence several aspects of the host biology, such as host energy metabolism (Gill et al., 2006), immunity (Round and Mazmanian, 2009; Kohl, 2012; Maynard et al., 2012; Kamada et al., 2013), development (Borre et al., 2014; Videvall et al., 2019; Kirschman et al., 2020), behavior (Archie and Theis, 2011; Ezenwa et al., 2012; Lim et al., 2016; Davidson et al., 2018, 2020; Johnson and Foster, 2018; Sherwin et al., 2019), and evolution (Moeller and Sanders, 2020). Therefore, taxonomic diversity of the microbial species contained in the gut, as well as their relative abundance is likely deterministic for the host’s fitness (Gould et al., 2018; Videvall et al., 2020).

The composition and the structure of gut microbial communities are shaped by the combination of extrinsic and host-specific factors in both stochastic and deterministic ways. Animals acquire their microbes vertically from their parents (Palmer et al., 2007; Jiménez et al., 2008; Collado et al., 2012; DiGiulio, 2012; Jeurink et al., 2013; Trevelline et al., 2018) or horizontally from the physical and social environment, first in the postnatal phases and then through their lives (Tung et al., 2015; Moeller et al., 2018; Chen et al., 2020). Therefore, the environmental pool of microorganisms and ecological parameters driving the distribution of microbial communities have non-negligible roles in shaping host-associated microbial communities (Candela et al., 2012; Rothschild et al., 2018). Besides, gut microbiota, – at least to some extent – is an entity, regulated by the host itself in a deterministic way. When an animal host cross paths with a microorganism, the host either selectively establishes symbiotic associations or eliminates the survival and reproduction of the microorganism. Although the exact mechanism of these complex processes is still largely elusive, it probably involves selective pressures imposed by distinct features of the gastrointestinal environment, physiology, immunity, and dietary adaptations of the host (Ley et al., 2008; Muegge et al., 2011; Kubinak et al., 2015; Stagaman et al., 2017). Consequently, it is feasible to assume that each individual harbors a unique microbial assemblage reflecting both, the signatures of their interactions with the environment (Engel et al., 2020) and host-specific factors. At the same time, considering the vital functions of the microbes in several physiological processes of the hosts, the gut microbiota, or at least a subset of microorganisms should be conserved among the members of the same species. In line with these assumptions, human microbiota consists of some temporally stable microbial species that are highly adapted to their host, along with some transient taxa that respond quite rapidly to the dietary, developmental, and physiological alterations (Turnbaugh et al., 2007; Lozupone et al., 2012; Lloyd-Price et al., 2017). Interestingly, in this rapidly growing field, we are still lacking the knowledge to what extent microbial communities are similar between conspecifics, what drives inter-individual differences and if and how stable are communities across the distinct life-history stages of individual hosts in non-human species.

This information gap is specifically striking for avian taxa (Maraci et al., 2018). Existing knowledge indicates that the gut microbiota of birds is shaped primarily by external factors such as diet (Blanco et al., 2006; Waite and Taylor, 2014; Bodawatta et al., 2018; Kohl et al., 2018; Michel et al., 2018; Teyssier et al., 2020), local habitat (Hird et al., 2014; Waite and Taylor, 2014), or nesting environment (Teyssier et al., 2018; van Veelen et al., 2020). Gut microbial species can be transferred between parents and offsprings (Chen et al., 2020) and breeding pairs (Kreisinger et al., 2015; Ambrosini et al., 2019). Individuals from the same nest have a similar gut microbial composition (Lombardo et al., 1996; Ambrosini et al., 2019). However, it is unclear whether these congruences are originating from genetic relatedness or similarities in diet or environmental conditions. Although the link between the gut microbial profile and host taxonomy (Ruiz-Rodríguez et al., 2009, 2018; Waite and Taylor, 2014; Hird et al., 2015) and genetics (Banks et al., 2009; Zhao et al., 2013) have been demonstrated, evidence for the strength of these host-specific factors is equivocal. This can be explained by the fact that to date, most avian studies that investigate the determinants of the gut microbiota have been carried out on wild animals in natural conditions, except for the poultry. While undoubtedly important, the findings of those studies on host-specific factors are clearly mitigated by strong environmental and dietary effects. In this regard, studies conducted under captivity conditions where the diet and environmental conditions are standardized would allow us to understand how much of the microbial diversity is driven by deterministic host-specific factors.

The present study aimed to document interspecies and intraspecies differences in gut microbiota in two estrildid finches, the zebra finch (*Taeniopygia guttata*) and the Bengalese finch (*Lonchura striata domestica*), under strictly controlled environmental and dietary conditions to determine whether gut microbial diversity is influenced by host-specific factors. If the gut microbiota is influenced by host taxonomy and genetics, we expect that (i) individuals of the same species should harbor more similar microbial profiles compared to members of different species, (ii) there should be consistent between-individual variations, and (iii) at least some proportion of the microbial species harbored by the hosts should be conserved over time, even throughout the different stages of the reproductive cycle. To test these assumptions and explore the impact of sex and social transmission, we characterized gut microbial profiles in breeding pairs of the zebra finches and the Bengalese finches at five different time points, over a period of 120 days.

## Materials and Methods

### Ethics Statement

Housing and breeding of birds were approved by the Gesundheits-, Veterinär-und Lebensmittelüberwachungsamt der Stadt Bielefeld (#530.421630–1,18.4.2002). All birds remained in the aviary stock after experimentation. All experiments were performed following the animal experimentation guidelines and laws of Germany.

### Study Organisms and Sampling

Between January and August 2017, we examined gut microbial profiles of 42 individuals of two captive bird species, the zebra finches (ZF; *N* = 28, 14 females, 14 males, referred to as “Bielefeld” in Forstmeier et al. (2007) and the Bengalese finches (BF; *N* = 14, 7 females, 7 males), from the laboratory stock at the Bielefeld University. The zebra finches were transferred from single-sexed indoor aviaries (2.30 × 2.90 × 3.30 m) to indoor cages (0.80 × 0.30 × 0.40 m) as pairs. The Bengalese finches were transferred from mixed-sex indoor aviaries (2.30 × 2.90 × 3.30 m) to indoor cages (0.80 × 0.30 × 0.40 m). After a week of habituation, nesting material (coconut fiber) was provided and a wooden nest box (15 × 15 × 15 cm) was attached at each cage. The pairs were kept in these cages until their youngest offspring reached nutritional independence (approximately 35 days after hatching). After this point, all birds were transferred into mixed-sexed indoor aviaries (2.30 × 2.90 × 3.30 m), where they were kept with other conspecifics from this study. All birds kept under a 14:10 h light/dark cycle with a temperature range of 24.5–25.5°C. They were fed the standard diet, comprising of seeds *ad libitum*, a vitamin–mineral supplement and additional egg food (Tropical Finches, CéDé, Evergreen, Belgium) and germinated seeds every day.

Previous studies showed that fecal sampling is a non-lethal and non-invasive method that can successfully capture gut microbial structure in different bird species (for example Videvall et al., 2018), including the zebra finches (Berlow et al., 2020). Therefore, we used community profiles from fecal samples as a proxy for the gut microbial communities. To collect feces, we transferred the birds to sampling cages (30 × 40 × 30 cm) where they were kept individually for 30 min. Before the sampling, cages were cleaned using 78% ethanol and the bottom of cages were covered with sterile aluminum plates. After 30 min, we transferred the fecal materials from the aluminum plates to sterile 2 ml Eppendorf tubes using a sterile pipet tip. The samples were kept at ice during the collection and directly transferred to −80 within the first hour after the collection, where they were stored until further processing. The sampling equipment was always handled using nitrile gloves sterilized by 78% ethanol. We collected samples at five different time-points during the breeding period (over 120 days). The first samples were collected during incubation, approximately 7 days after the completion of the clutch. The second and third samples were collected during the chick-rearing period, 5 and 10 days after hatching of the youngest chick, respectively. The fourth samples were collected when the juveniles reached nutritional independence, 35 days after hatching of the youngest chick. The fifth samples were collected after the reproductive phase, when the juveniles reached sexual maturity, 100 days after hatching of the youngest chick. In total, we collected 210 samples from 42 birds.

### DNA Extraction and Library Preparation

Microbial DNA was extracted from 0.02 grams of the fecal sample using the QIAamp PowerFecal DNA Kit (Qiagen), according to the manufacturer’s instructions. We prepared the16S rRNA gene libraries following the Illumina 16S Metagenomic Library Preparation Guide, 15044223-B. We targeted hypervariable V3–V4 region of the 16S ribosomal RNA (rRNA) genes by using the primers 5′-CCTACGGGNGGCWGCAG-3′ and 5′-GACTACHVGGGTATCTAATCC-3′ (Klindworth et al., 2013). The Illumina overhang adapters attached to the primers were as follows:

Forward: 5′-TCGTCGGCAGCGTCAGATGTGTATAAG AGACAG-3′,
Reverse: 5′-GTCTCGTGGGCTCGGAGATGTGTATAAG AGACAG-3′.

The first polymerase chain reactions (PCR) was performed in a 25 μL reaction volume containing 5 μL DNA, 12.5 μL KAPA HiFi HotStart ReadyMix (KAPA Biosystems, MA, United States), 2.5 μL of each primer (2 μM) and 5 μL of PCR grade water. The amplification conditions were as follows: an initial denaturation step at 95°C for 3 min, followed by 25 cycles of denaturation at 95°C for 30 s, annealing at 55°C for 30 s, extension at 72°C for 30 s, with a final extension step of 5 min at 72°C. To remove free primers and primer dimers and PCR products were subsequently purified using the Agencourt AMpure XP PCR purification system (Beckman Coulter, Brea, CA, United States) as described in the manufacturer’s protocol. To be able to multiplex the libraries, Dual Illumina indices (The Nextera XT Index Kit, Illumina, Inc., San Diego, CA, United States) were attached to the PCR products were by another PCR which was performed in a 50 μL volume containing 5 μL of the purified amplicon PCR product, 25 μL KAPA HiFi HotStart ReadyMix, 5 μL of each index primer, 10 μL of PCR grade water. The temperature profile of the amplification was as follows: an initial denaturation step at 95°C for 5 min, followed by eight cycles of 30 s at 95°C, 30 s at 55°C 30 s at 72°C with a final extension step of 5 min at 72°C. Two blank controls for PCR amplification and five replicates of the same sample were also included in the sequencing. The amplified fragments were subjected to another purification step using the Agencourt AMpure XP PCR purification system. The size of the amplified fragments was verified by running 1 μl of 1:50 dilutions of the final libraries on a Bioanalyzer DNA 1000 chip (Agilent Technologies, Palo Alto, CA, United States). Then, the concentration of the libraries was quantified by PicoGreen dsDNA Assay on a TECAN Infinite Reader M200 instrument. Seven samples were discarded due to unsuccessful amplification. Three negative control of extraction, PCR and clean-up steps and two technical replicated of three samples were included in the final pool. Accordingly, the final library contained equal concentrations of 209 uniquely barcoded amplicons. Sequencing of the final library was performed using paired-end mode (2 × 300 sequencing cycles) on the Illumina MiSeq system (Illumina, Inc., San Diego, CA, United States) at the CeBiTec, Bielefeld University.

### Bioinformatics Processing

Processing of raw MiSeq forward and reverse PE reads were done as described by Engel et al. (2018) with the following minor adjustments to individual bioinformatic steps. To achieve a higher assembly rate, we assembled Miseq PE reads in an iterative manner using Flash v1.2.11 (Magoč and Salzberg, 2011). All reads failing the first round of read assembly were clipped to a q20 average quality threshold using sickle v1.33 (Joshi and Fass, 2011) and re-submitted to flash. This process was consecutively repeated while increasing the quality clipping threshold by three up to the point where either all reads could be assembled or a maximum quality clipping threshold of q35 was reached. All other steps, i.e., (i) adapter clipping with cutadapt v1.18 (Martin, 2011), (ii) de-replication, alignment, filtering, and de-noising with mothur v1.41.3 (Schloss et al., 2009), (iii) chimera checking and operational taxonomic unit (OTU) clustering with USEARCH v8.0.1477 (Edgar, 2010), and (iv) taxonomically classification based on the full SILVA database v138 (Quast et al., 2013) was carried out as described in detail previously (Engel et al., 2018) but without performing a length trimming step after primer clipping. The reason for employing the OTU approach is that we are mainly interested in structural community differences or conformities rather the detection of extremely rare and low abundant community members. Therefore, the finer taxonomic resolution provided by ASVs over OTUs does not further our understanding.

### Statistical Analyses

We conducted all consecutive statistical analyses in R version 4.0.0 (R Core Team, 2020) and Primer-e software version 7 (Clarke et al., 2014). The code used in this study is provided in the GitHub repository at https://github.com/AnnaAntonatouPap/Microbiota-of-estrildid-finches-.git. We excluded the samples with less than 10,000 total read counts (*n* = 5). After this filtering step, 204 samples were retained in the dataset. We filtered out all the OTUs that could not be classified at phylum level or that were classified as mitochondria or chloroplasts (*n* = 44) as those are very likely to be issued by sequencing errors.

To account for the potential bias due to the uneven sequencing depth across the samples, we rarefied OTU read count data to the lowest read count observed in the dataset (12,472) and estimated alpha diversity based on this dataset. We computed Shannon’s diversity index, which accounts for both abundance and evenness of the taxa present (Shannon, 1997). We investigated the drivers of alpha diversity using a linear mixed model, as implicated in the lme4 package version 1.1−15 (Bates et al., 2015). We used untransformed Shannon’s diversity index as the response variable; the host ID and couple ID as random effects; species, sex, and sampling time as fixed effects. We also included the interaction between sex and sampling time as fixed effects to account for potential sex-specific changes in alpha diversity over time. Residuals of the models were inspected visually. To visualize the taxonomic and compositional structure of the microbial communities in the zebra finches and the Bengalese finches based on the non-rarefied dataset, we produced stacked bar plots, based on the family level taxonomy using ggplot2 version 3.3.2 (Wickham, 2009).

To estimate between-group diversity, first, the filtered dataset was subjected to Cumulative Sum Scaling (CSS) normalization (Paulson et al., 2013a) using the r package metagenomeseq version 1.30.0 (Paulson et al., 2013b) to deal with unequal sequence coverage. Subsequently, to account for compositional variations within the data, we Log (*x* + 0.0001)−transformed the data and later corrected the transformed values by subtracting the log of the pseudo count as recommended by Thorsen et al. (2016). Then, we computed a dissimilarity matrix based on Jaccard (1912), Bray–Curtis (Bray and Curtis, 1957), unweighted UniFrac (Lozupone and Knight, 2005), and weighted UniFrac (Lozupone et al., 2007) distances. To visualize the dissimilarities between the zebra finches and the Bengalese finches, we used a principal coordinate analysis (PCoA) as implemented by the function “ordinate” in vegan package version 2.5-6 (Oksanen et al., 2019). Based on PCoA plots, we selected the two dissimilarity measures explaining the highest variation (Bray–Curtis and weighted UniFrac) and used these distance matrices in all further analyses. We statistically tested the differences in the gut microbial assemblages in *a priori* defined groups by non-parametric analysis of similarities (ANOSIM) (Clarke, 1999) with 999 permutations in Primer 7. We analyzed the differences in microbial communities between the host species, performing one-way ANOSIM. We tested whether the samples collected from the same individual are more similar to each other compared to the samples collected from different individuals using two-way nested ANOSIM (individuals nested in species). To further evaluate whether the length of the period between the sample collection has any effect on microbial composition, we calculated a distance matrix that incorporated the time between the sample collection (setting the first sampling = 0). Then we performed a Mantel-like test (RELATE) to analyze the correlation between the distance matrix based on weighted UniFrac and the distance matrix of the time between sample collection by correcting for host ID, using Primer 7. We analyzed whether the individuals of the same sex have more similar microbial communities using a two-way crossed ANOSIM. Next, to address the potential sex-specific temporal differences in the community structure, we subset the samples collected from a given sex of each species. In each group, we tested whether the samples collected at a particular time point are more similar then the samples collected at different time points using one-way ANOSIM. Finally, to evaluate whether the mating pairs have more similar microbial communities compared to randomly chosen males and females, we first merged the samples collected from the same individuals by averaging taxonomic representatives from these samples to prevent any bias due to the use of repeated sampling of the same bird. Second, we performed a two-way nested ANOSIM (couples nested in species).

To identify specific OTUs potentially shaping the differences among species, we analyzed differentially abundant OTUs. We estimated logarithmic fold changes between groups by a negative binomial Wald test as implemented in DESeq2 version 1.12.4 extension (Love et al., 2014) of the Phyloseq package version 1.32.0 (McMurdie and Holmes, 2013). As a significance threshold for *p*-values, we used a 0.01 threshold after a Benjamini and Hochberg (1995) false−discovery rate correction.

## Results

We sequenced the hypervariable V3–V4 region of the 16S rRNA gene from 210 gut microbial community samples originating from 42 individual birds. After the bioinformatics processing, our dataset consisted of 209 samples, 626 different OTUs with a total read count of 14,821,860 (mean: 72,656). After the filtering process, our dataset contained 204 samples, 582 OTUs with a total read count of 14,639,903 (Mean = 71,764.23).

**FIGURE 1:**
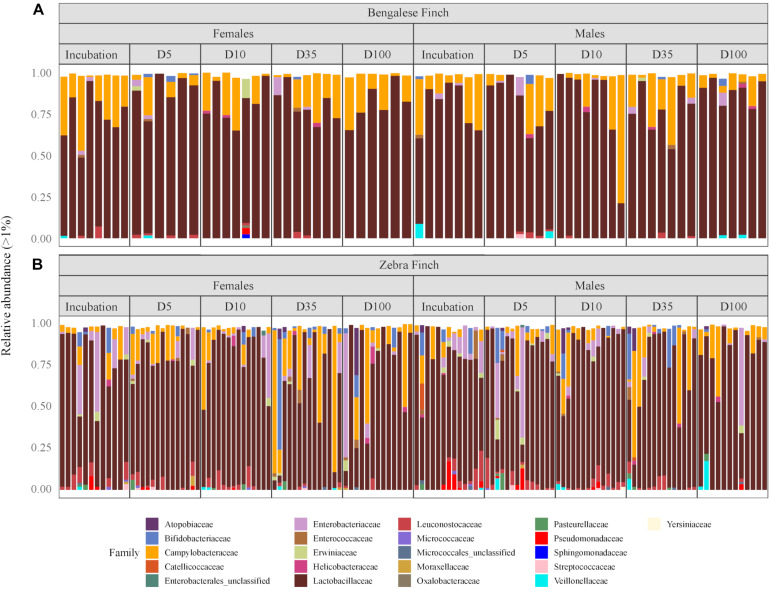
The relative abundance of microbial families in gut samples from **(A)** the Bengalese Finches and **(B)** the zebra finches through the different phases of the reproductive cycle: at incubation, 5 days (D5), 10 days (D10), 35 days (D35), and 100 days (D100) after hatching the youngest chick, respectively. Rare phyla with relative abundances below 1% are not shown.

We identified 20 microbial phyla, with the domination of Firmicutes (79.15%), Campilobacterota (14.07%), Proteobacteria (4.36%), and Actinobacteria (2.40%) (the numbers indicate the total abundance; the mean values and standard deviations are provided in Supplementary Table 1). At a finer taxonomic scale, identified taxa corresponded to 199 microbial families, 96.26% of which is constituted by Lactobacillaceae (76.10%), Campylobacteraceae (13.63%), Enterobacteriaceae (3.03%), Leuconostocaceae (1.88%), and Bifidobacteriaceae (1.62%) (Supplementary Table 2). Although the most abundant families remained the same through our study, we observed some compositional alterations among the species and across time (Figure 1).

### Structure of the Gut Microbial Communities in Relation to Host Taxonomy

**FIGURE 2:**
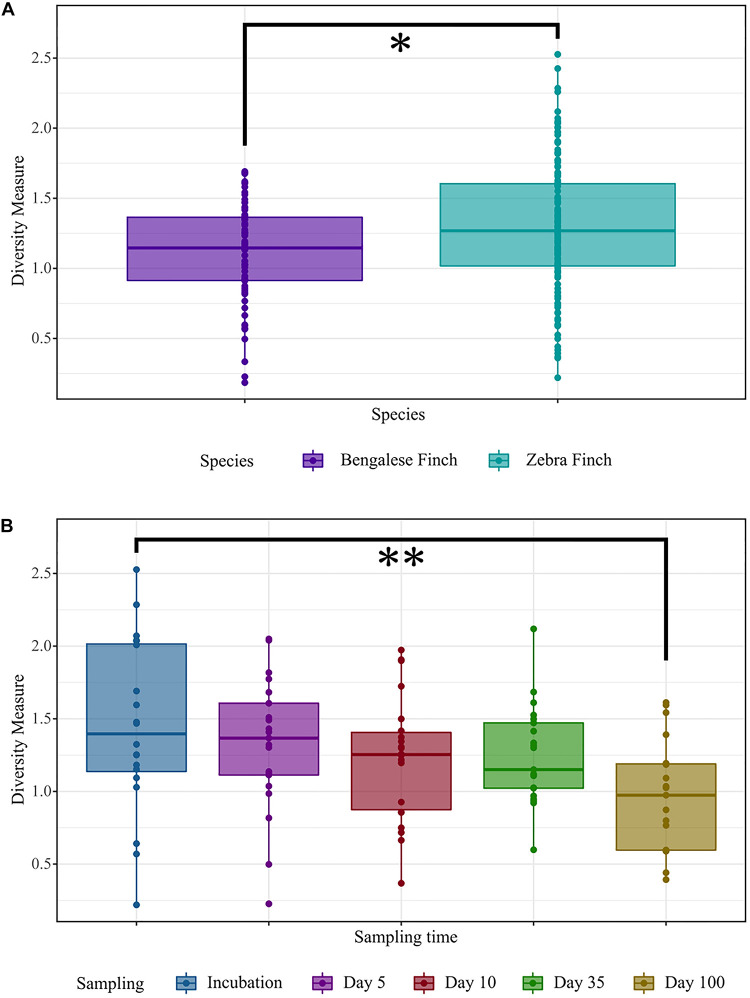
Comparisons of Shannon’s diversity index. **(A)** Among the host species (Bengalese Finches and zebra finches). **(B)** In males of both species across five different sampling times: at incubation, 5 days (D5), 10 days (D10), 35 days (D35), and 100 days (D100) after hatching of the youngest chick, respectively. The significance was determined based on the linear mixed model, at *p*-values ≤ 0.05 (*), *p* ≤ 0.01 (**), and *p* ≤ 0.001 (***). In the box plots, the line within indicates the median and the lower and upper boundary of the boxes indicates the 25th and 75th percentile, respectively. Whiskers above and below the boxes correspond to the range of 1.5 times the inter-quartile range (IQR) above and below the 25th and 75th percentile, respectively.

We found pronounced differences in the gut microbial structure of the zebra finches and the Bengalese finches. Based on the linear mixed model, which explained a considerable variation in diversity (*R*^2^-marginal = 0.117, *R*^2^-conditional = 0.261), we found a significant difference in the alpha diversity estimates between the two bird species: the zebra finches exhibited higher diversity based on Shannon diversity index (β = 0.19 ± 0.09, 95% CI [0.02 − 0.35], *p* = 0.028; Figure 2A).

**FIGURE 3:**
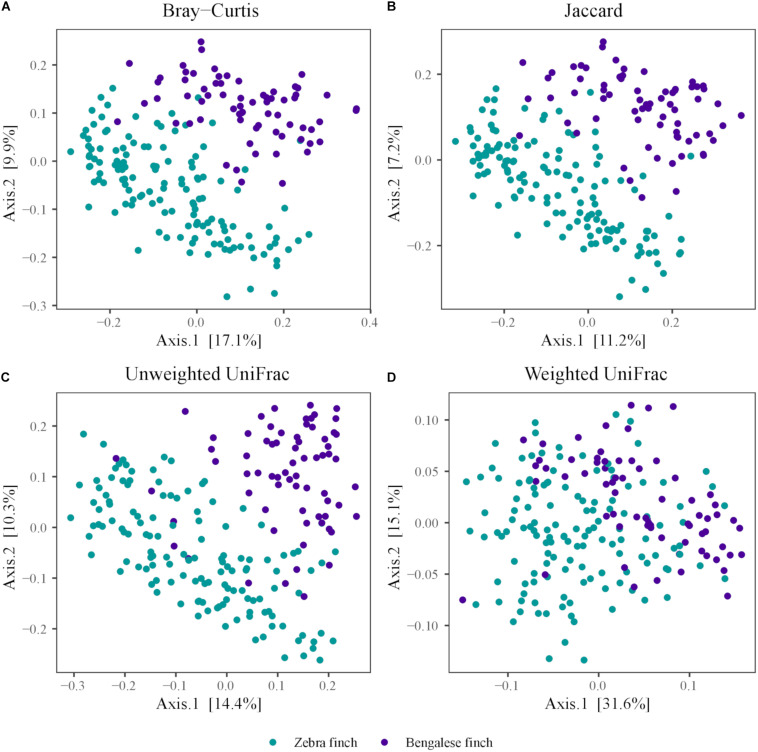
Principal coordinate analysis plots of the dissimilarities of zebra Finch and the Bengalese finch gut microbiota. Distances were computed using the **(A)** Jaccard and **(B)** Bray–Curtis dissimilarity index, and the **(C)** unweighted **(D)** weighted UniFrac distance metric.

We visualized the dissimilarities in microbial communities among the species using a PCoA based on Jaccard (Figure 3A), Bray–Curtis (Figure 3B), unweighted (Figure 3C), and weighted UniFrac distances (Figure 3D). The microbial communities harbored by each species clustered together in all plots. As the variation explained by the first two axes was higher in the PCoA plots based on Bray–Curtis (Axis1: 17.1%, Axis2: 9.9%) and weighted UniFrac distances (Axis1: 32%, Axis2: 15.2), compared to Jaccard (Axis1: 11.2%, Axis2: 7.2%) and unweighted UniFrac (Axis1: 15%, Axis2: 10.6%) dissimilarity, we used the former two when computing ANOSIM to statistically test for potential differences between the two species. The ANOSIM confirmed a statistical difference in the microbial communities between the two species, which was already revealed by PCoA (based on Bray–Curtis; one-way ANOSIM; factor *species*: global *R* = 0.247, *p* = 0.0001; based on weighted UniFrac, one-way ANOSIM; factor *species*: global *R* = 0.230, *p* = 0.0001).

**FIGURE 4:**
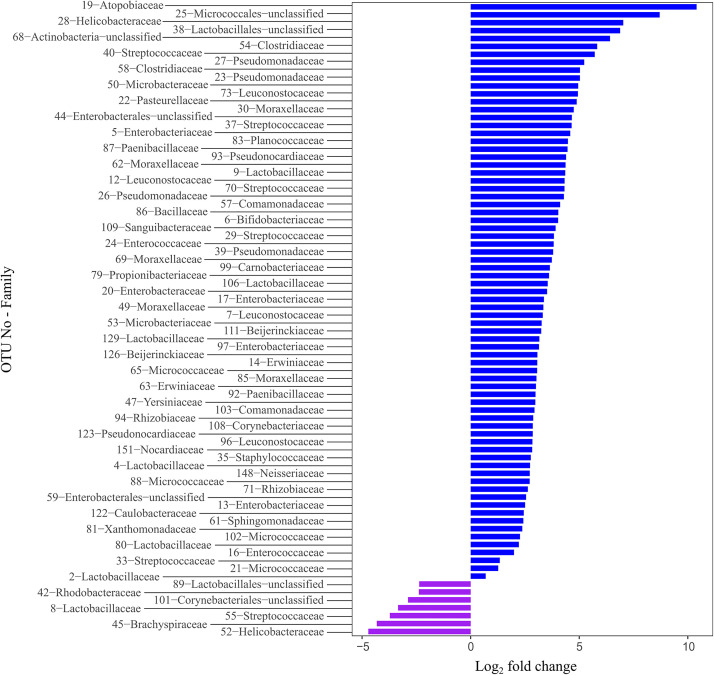
The differentially abundant phyla between the zebra finches and the Bengalese finches. Bars represent OTUs that are significantly differentially abundant between the two host species. OTUs with a log_2_–fold–change larger than zero are more abundant in the zebra finches (blue bars), while the OTUs with a log_2_–fold–change smaller than zero are more abundant in the Bengalese finches (purple bars).

We determined the differentially abundant OTUs underlying the observed differences among the species using the DESeq2 method. We provided detailed information on differentially abundant OTUs in the Supplementary Table 3. Overall, we found 81 significantly differentially abundant OTUs, which constituted for 13.91% of all OTUs (Figure 4). Of these, seven were significantly more abundant in the Bengalese finches and 74 were significantly more abundant in the zebra finches. Expect three of those differentially abundant OTUs, all belonged to three microbial phyla: Proteobacteria (*n* = 32), Firmicutes (*n* = 29), and Actinobacteriota (*n* = 17).

### Structure of the Gut Microbial Communities in Relation to Host ID

The microbial composition of samples collected from the same individual (Defined by the *Host ID*) at different time points were on average more similar than those of the samples collected from different individuals (two-way nested ANOSIM Bray–Curtis; Individuals nested in species; factor *species*: global *R* = 0.442, *p* = 0,001; factor *Host ID*: global *R* = 0.273, *p* = 0.001; Weighted UniFrac, factor *species:* global *R* = 0.527, *p* = 0.001; factor *Host ID*: global *R* = 0.096, *p* = 0.001; Supplementary Figure 1A). Furthermore, in zebra finches the length of the period between the sample collection negatively correlated with the microbial similarity: the smaller the sampling periods, the more similar the microbial communities are (Mantel test, Rho: 0.142, *p* = 0.038). However, this correlation was not evident in the Bengalese finches (Mantel test, Rho: 0.085, *p* = 0.181).

### Sex-Specific Differences in Diversity and Structure of the Gut Microbial Communities

When we analyzed the alpha diversity in relation to host sex, we did not find any evidence for sex-specific differences (the linear mixed model, β = 0.04 ± 0.13, 95% CI [−0.21 to 0.30], *p* > 0.5). Furthermore, no significant differences were found in the microbial communities between males and females of a given species (two-way crossed ANOSIM; Bray–Curtis; factor *Species*: global *R* = 0.246, *p* = 0.001; factor *Sex*: global *R* = 0.004, *p* = 0.263; Weighted UniFrac; factor *Species*: global *R* = 0.229, *p* = 0.001; factor *Sex*: global *R* = −0.004, *p* = 0.673). However, as the linear mixed model showed a reduction in alpha diversity over time (β = −0.25 ± 0.12, 95% CI [−0.49 to −0.01], *p* = 0.038), we partitioned the diversity across sexes. In males, the microbial diversity decreased over time with a significant difference between incubation and day 100 (β = −0.48 ± 0.12, 95% CI [−0.87 to −0.08], *p* = 0.005; Supplementary Figure 1B). By contrast, we did not observe any significant diversity change across sampling times in the females. When comparing the gut microbial communities across the time within a given sex of each species, we found that both taxonomic composition and relative abundance of the microbial assemblages is changing over time in the zebra finch males (ANOSIM; Bray–Curtis; global *R* = 0.115 *p* = 0.0003; Weighted UniFrac; global *R* = 0.067, *p* = 0.009; Supplementary Figure 1B). In the Bengalese finch males, the alterations between the sampling times were only significant when using the Bray–Curtis measure (global *R* = 0.118 *p* = 0.009; Supplementary Figure 1B). No significant change was observed in females.

### Microbial Similarity Between Mating Pairs

Mating pairs (defined by the *Couple ID*) had more similar microbial assemblages compared to randomly chosen males and females (two-way nested ANOSIM; *Couples* nested in *species*, Bray–Curtis; factor *species*: global *R* = 0.679, *p* = 0.001; factor *Couple ID*: global *R* = 0.555, *p* = 0.001; Weighted UniFrac; factor *species*: global *R* = 0.54, *p* = 0.001; factor *Couple ID*: global *R* = 0.115, *p* = 0.001; Supplementary Figure 1C).

## Discussion

The relevance of symbiotic microorganisms for host ecology and evolution has become increasingly evident. However, we still lack a full understanding of the factors shaping microbial assemblages. Although studies are accumulating, the knowledge on microbial symbiosis in birds is still far behind compared to mammalian species. Furthermore, a huge proportion of the avian studies are conducted on wild populations in their natural habitats (reviewed in Maraci et al., 2018), where several environmental parameters and host-specific factors interact together to shape patterns of microbial colonization and maintenance. Although these studies are very important, they provide little insight into the relative contribution of host-specific factors. Controlled studies enable us to comprehend to what extent hosts can control their microbial assemblages by minimizing the impact of host diet and other extrinsic factors. However, such studies are scarce in avian species. To fill this gap, we characterized gut microbial assemblages of the zebra finches and the Bengalese finches under strictly controlled conditions. As expected, we found prominent interspecific differences in the structure of gut microbial communities. Although the individuals exhibited unique, individual-specific microbial patterns over 3 months, males of both species manifest higher within-individual variations, compared to females. Our study conclusively shows that gut microbes are host-specific, providing essential insights on the host characteristics shaping gut microbial communities.

### Taxonomic Composition of the Gut Microbial Communities

Microbial assemblages of the zebra finches and the Bengalese finches were dominated by four microbial phyla: Firmicutes, Campilobacterota, Proteobacteria, and Actinobacteria. The microbial structure recovered in our study was remarkably different from that of wild birds (Hird et al., 2015; Grond et al., 2018). Firmicutes were overrepresented, while there is a deficit of proteobacteria in our study, which is consistent with the findings of previous studies carried out under captivity (Xie et al., 2016). In our study, the second most abundant phylum was Campilobacterota, which is not a usual phylum reported by avian studies conducted under natural or captivity conditions. However, the phylum Campilobacterota was introduced with the SILVA database v138 release, replacing the phylum Epsilonbacteraeota, which was indeed among the abundant phyla reported in previous avian studies (Zhao et al., 2019). This change to the SILVA reference taxonomy, among other taxonomic changes on various levels (York, 2018), was done as SILVA now adopts to the Genome Taxonomy Database (GTDB), a new and more precise taxonomy based on phylogeny inferred from the concatenation of 120 ubiquitous single-copy proteins (Parks et al., 2018). Although some members of this phylum can potentially cause diseases in wild and domestic animals (Bull et al., 2008), they are considered to be non-pathogenic in bird species (Oakley et al., 2014) and are frequently isolated from healthy zebra finches (Benskin et al., 2010; Chen et al., 2020).

### Species-Specific Differences in Community Diversity and Composition

A substantial body of evidence has revealed that host taxonomy and evolutionary history are among the main determinants of the gut microbial communities harbored by mammals (Ochman et al., 2010; Phillips et al., 2012; Moeller et al., 2014; Knowles et al., 2019; Song et al., 2020). However, the avian studies investigating interspecific differences in host-associated microbial communities revealed contrasting findings. Some studies failed to show a link between host-taxonomy and gut microbial structuring (Ruiz-Rodríguez et al., 2009, 2018; Hird et al., 2014). On contrary, some comparative studies have shown remarkable species-specific differences (Waite and Taylor, 2014; Hird et al., 2015; Michel et al., 2018; Fu et al., 2020; Lee et al., 2020). It is, however, important to note that all these studies have been conducted using natural populations. As different bird species have distinct dietary preferences and occupy different habitats with altering ecological variables it is not clear whether the detected patterns reflect a true host-specificity.

In the present study, although some proportion of the gut microbiota was conserved between the zebra finches and the Bengalese finches, we also found remarkable differences in the gut microbial communities between these two closely related bird species. Zebra finches harbored more diverse microbial colonies compared to the Bengalese finches. The microbial communities of the two species exhibited variations in terms of taxonomic diversity, relative abundance, and phylogenetic distance of the harbored species. As the diet and other environmental parameters were strictly controlled in our experiment and the birds were housed in the same environment and consequently exposed to the same microbial reservoir, these findings likely underpin the differences in host biology. Although Bengalese finches and zebra finches are widely used in the ecological investigations, interestingly the studies investigating biological differences between these two species are limited to vocal communication, impeding any conclusive inference on what drives species-specific differences in microbial communities. However, one potential explanation for the observed changes is interspecific differences in physiology and anatomy of the digestive system. Although the gross anatomy of the digestive system is expected to be similar in these two bird species, there can be fine-scale anatomical and physiological differences which were indicated by two studies showing that mechanics of drinking movement (Heidweiller and Zweers, 1990) and water retention efficiency (Gere, 1977) differs between Bengalese finches and zebra finches. Alternatively, given the fact that host genetic variation, especially the allelic diversity of immune genes such as immunoglobulin genes, major histocompatibility (MHC) genes, toll-like receptors and cytokines, affects diversity and community structure of host-associated microbes (Bolnick et al., 2014; Blekhman et al., 2015), it is feasible to assume that observed microbial differentiation might reflect prominent genetic differences among the species. Considering the ancestral species White-rumped munia (*Lonchura striata acuticauda*) and wild zebra finch (*T. guttata*) originate from finches coming from two geographically distinct locations – China and Australia, respectively – presumably, they experienced differential selection pressures exposed by coevolving pathogens, which in turn result in variations in immune genes. Another possibility is that the strength of artificial selection during the domestication was different for these two species resulting in differential patterns of genetic variation. These predictions partially align with the existing literature: a comparative study investigating the genetic variability between captive and wild populations of zebra finches revealed that domestication led to the loss of some genetic variation but not a severe bottleneck (Forstmeier et al., 2007). Furthermore, domesticated zebra finches also exhibited high allelic diversity for the MHC complex class I genes which play a crucial role in adaptive immunity (Newhouse and Balakrishnan, 2015). To the best of our knowledge, there is no study investigating the loss of genetic diversity in immune genes due to the domestication Bengalese finches. However, considering during the domestication of Bengalese finches from White-rumped munias, breeders selected some specific traits such as good parenting behavior and white color morphs (Takahasi and Okanoya, 2010), presumably domestication led to a substantial decrease in host genetic diversity. However, all these potential interactions warrant further testing.

### Individual-Specificity and Stability of Microbial Communities

In humans, tremendous inter-individual variations in microbial communities were documented (Turnbaugh et al., 2009; Lozupone et al., 2012; Kolde et al., 2018) and some of these variations were shown to be stable over time (Lozupone et al., 2012; Faith et al., 2013) leading to the conclusion that individuals have unique microbial profiles. Interestingly, these inter-individual variations in the gut microbial repertoire are prominent shortly after birth (Dominguez-Bello et al., 2010; Raveh-Sadka et al., 2015), while the individuals have very limited interactions with their environment, suggesting the role of vertical transmission and host genetics in shaping individual-specific microbial profiles. Nevertheless, far less is known about the individual-specificity of microbial communities in non-human species.

Temporal stability of gut microbes has been studied in zebra finches using temperature gradient gel electrophoresis (Benskin et al., 2010). However, to the best of our knowledge, individual specificity, and long-term stability of gut microbiota in avian species under controlled conditions were investigated using a metagenomic approach for the first time in our study. We found significant inter-individual variations in microbial communities. The samples collected from the same individual were more similar to one another than those from different individuals. Although we observed some temporal variations in the composition of gut microbial communities in males (as explained in more detail in the next section), a considerable proportion of the gut microbes harbored by individuals was conserved throughout our study. These findings are consistent with the previous study (Benskin et al., 2010) and emphasize the idea that some microbial species are highly adapted to their host and resilient to perturbations, while others are more flexible (Lozupone et al., 2012; Ursell et al., 2012). Accordingly, it is feasible to conclude that hosts can, at least to some extent, control their microbial communities. This further indicates that host genetics has a function in structuring gut microbial communities. However, the exact genetic pathways involved in the regulation of host-symbiont interactions and consequences of these inter-individual variations warrants further investigation.

### The Effect of Sex and Sex-Specific Temporal Alterations in Community Diversity and Composition

As males and females of a given species have some differences in their behavior, hormonal profiles and physiology (Tarka et al., 2018), which might influence their symbiotic interactions, we would expect to observe some microbial differences among the sexes of the same species. However, the evidence for the impact of sex on microbial communities is equivocal. In some species, there are sex-specific differences (Leclaire et al., 2014; Elderman et al., 2018; Stoffel et al., 2020), while in others no sexual dimorphism was detected (Benskin et al., 2010; Tung et al., 2015; Bennett et al., 2016; Ren et al., 2017).

In our study, there were no differences in alpha and beta diversity between males and females of the host species, when analyzed regardless of sampling time. However, when we examined the temporal changes in alpha and beta diversity across sexes, we observed sex-specific differences: the males exhibit temporal alterations in both alpha and beta diversity indices, while these metrics remained the same in females, over time. The observed sexual dimorphism can be explained by the plastic alterations in gut microbial communities in response to fluctuating testosterone levels during reproduction. Our sampling times coincide with the different stages of the reproductive cycle, covering all phases between incubation and post-reproduction. Testosterone mediates several reproductive behaviors (Adkins-Regan, 2005) and suppresses the immune system (Folstad and Karter, 1992). This suppression can allow the opportunistic microorganisms to colonize different habitats in the animal body and lead to an increased microbial diversity (Escallón et al., 2017). In socially monogamous birds with biparental care, like the zebra finches or the Bengalese finches, testosterone levels peak during the mating phase declines during the parental care phase with fluctuations and reaches lowest levels during the non-breeding period after reproduction (Hill et al., 2005; Schwabl et al., 2005). In our study, alpha diversity followed the same pattern in males. They were highest during incubation, the phase just after mating, decreased during parental care phase and lowest during non-breeding. Similarly, in rufous-collared sparrows (*Zonotrichia capensis*), cloacal microbial communities exhibited sexual dimorphism. In males, the diversity decreased during reproduction and increased again as they transitioned to non-breeding condition, while this pattern has not been observed in females (Escallón et al., 2019). However, as we did not measure the testosterone levels in our study, this interaction remains to be investigated.

### Microbial Similarity Between the Mating Pairs

We observed similarities between the microbial compositions of mating pairs. This finding supports the existing body of literature on transmission of cloacal bacteria among sexual partners in birds (Kulkarni and Heeb, 2007; White et al., 2010; Escallón et al., 2019). A birds’ cloaca performs multiple functions for the digestive, urinary, and reproductive system. During sexual intercourse, it allows the transfer of microbes between the pairs, from these different sources. Although this kind of microbial exchange can lead to the transmission of diseases, it can also ensure the transfer of beneficial microbes between sexual partners (Smith and Mueller, 2015). Potential fitness benefits provided by sexually transmitted microbes and involvement of these microbes into mate choice decisions should be further studied.

## Conclusion and Outlook

Symbiotic microorganisms influence nearly all aspects of an animals’ biology. However, we still know very little about the role of host characteristics in the regulation of these complex systems, especially in avian taxa. This is partly because most studies have been conducted on wild populations, and in such systems, host-specific factors can be masked by spatially varying environmental factors. On the contrary, our study was conducted under fully controlled conditions to minimize interference of dietary and environmental variations and therefore has specific importance in terms of understanding the effect of host-specific factors in sculpturing symbiotic microbial assemblages. Our study convincingly shows that in zebra finches and Bengalese finches gut microbes are species-specific, unique to individuals, considerably stable over-time (with some lesser extent fluctuations in males across the different stages of the reproductive cycle) and exhibit similarities between mating pairs. This descriptive study is an important first step in elucidating the function of host-microbe interactions in avian ecology and evolution.

Our findings have naturally raised some further questions to be investigated. For example, an investigation of heritable components of the microbiota and the relative importance of different transmission routes can improve our understanding of the species-specificity of gut microbes. Furthermore, the exact mechanisms enabling the individual hosts to regulate this highly complex system warrant further research. In this regard, the investigation of host immune genes can broaden our insights about the colonization dynamics. Lastly, the interaction between reproductive hormones and gut microbial composition should be further studied.

## Data Availability Statement

The datasets presented in this study can be found in online repositories. The names of the repository/repositories and accession number(s) can be found below: European Nucleotide Archive, Project ID: PRJEB41573, accession number ERP125369.

## Ethics Statement

The animal study was reviewed and approved by Gesundheits-, Veterinär-und Lebensmittelüberwachungsamt der Stadt Bielefeld (#530.421630–1,18.4.2002).

## Author Contributions

ÖM and BC conceptualized the research idea and planned the experiments. ÖM carried out the experiments. TB contributed to the sample preparation. JK contributed to the analytical tools. SJ performed the bioinformatic analyses. AA-P and OC-G carried out the statistical analysis with the supervision of BC and ÖM. ÖM wrote the manuscript in consultation with BC. All authors approved the final version of the manuscript.

## Conflict of Interest

The authors declare that the research was conducted in the absence of any commercial or financial relationships that could be construed as a potential conflict of interest.

## Supplementary Material

Supplementary Figure 1 Principal coordinate analysis plots computed using Bray–Curtis dissimilarity index visualized by **(A)** Species and host ID. **(B)** Sex and sampling time. **(C)** Species and couple ID. In the three different versions of the same PCoA plot, different color, and shape schemes indicate different variables influencing beta diversity. In plots **(A)** and **(C)** the host species are represented by different shapes (zebra finches are represented by the circles and the Bengalese finches are represented by triangles) while color codes are used to indicate individual ID and couple ID, respectively. In plot **(B)**, host sexes are represented by different shapes (females are represented by squares and males are represented by stars) and the samples are color coded according to the sampling time.

Supplementary Table 1 The total abundance, the mean abundance and the standard deviation of the identified microbial phyla. The numbers represent the percentages.

Supplementary Table 2 The total abundance, the mean abundance and the standard deviation of the identified microbial families. The numbers represent the percentages.

Supplementary Table 3 Detailed information on the differentially abundant microbial taxa in the zebra finches and the Bengalese finches.
