# Supplementary figures and images for: The Gut Microbial Composition Is Species-Specific and Individual-Specific in Two Species of Estrildid Finches, the Bengalese Finch and the Zebra Finch
Source: Front Microbiol. 2021 Feb 19;12:619141. doi: 10.3389/fmicb.2021.619141 (PMC7933042; doi:10.3389/fmicb.2021.619141)

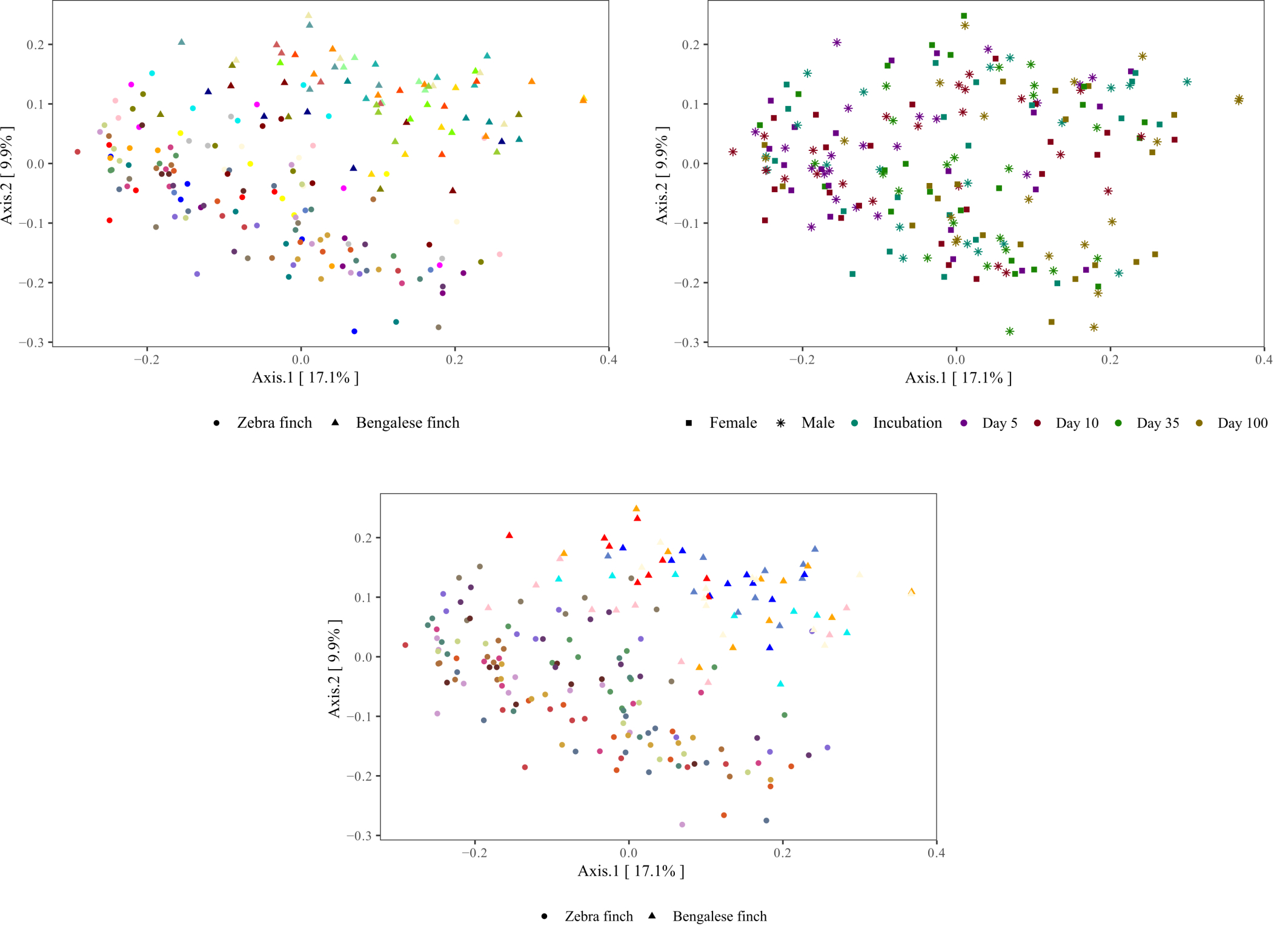

Supplement: Supplementary Figure 1 — Principal coordinate analysis plots computed using Bray–Curtis dissimilarity index visualized by (A) Species and host ID. (B) Sex and sampling time. (C) Species and couple ID. In the three different versions of the same PCoA plot, different color, and shape schemes indicate different variables influencing beta diversity. In plots (A) and (C) the host species are represented by different shapes (zebra finches are represented by the circles and the Bengalese finches are represented by triangles) while color codes are used to indicate individual ID and couple ID, respectively. In plot (B), host sexes are represented by different shapes (females are represented by squares and males are represented by stars) and the samples are color coded according to the sampling time. [file Image_1.JPEG]
